# Enhanced O-glycosylation site prediction using explainable machine learning technique with spatial local environment

**DOI:** 10.1093/bioinformatics/btaf034

**Published:** 2025-01-29

**Authors:** Seokyoung Hong, Krishna Gopal Chattaraj, Jing Guo, Bernhardt L Trout, Richard D Braatz

**Affiliations:** Department of Chemical Engineering, Massachusetts Institute of Technology, Cambridge, MA 02139, United States; Department of Chemical Engineering, Massachusetts Institute of Technology, Cambridge, MA 02139, United States; Department of Chemical Engineering, Massachusetts Institute of Technology, Cambridge, MA 02139, United States; Department of Chemical Engineering, Massachusetts Institute of Technology, Cambridge, MA 02139, United States; Department of Chemical Engineering, Massachusetts Institute of Technology, Cambridge, MA 02139, United States

## Abstract

**Motivation:**

The accurate prediction of O-GlcNAcylation sites is crucial for understanding disease mechanisms and developing effective treatments. Previous machine learning (ML) models primarily relied on primary or secondary protein structural and related properties, which have limitations in capturing the spatial interactions of neighboring amino acids. This study introduces local environmental features as a novel approach that incorporates three-dimensional spatial information, significantly improving model performance by considering the spatial context around the target site. Additionally, we utilize sparse recurrent neural networks to effectively capture sequential nature of the proteins and to identify key factors influencing O-GlcNAcylation as an explainable ML model.

**Results:**

Our findings demonstrate the effectiveness of our proposed features with the model achieving an $F_{1}$ score of 28.3%, as well as feature selection capability with the model using only the top 20% of features achieving the highest $F_{1}$ score of 32.02%, a 1.4-fold improvement over existing PTM models. Statistical analysis of the top 20 features confirmed their consistency with literature. This method not only boosts prediction accuracy but also paves the way for further research in understanding and targeting O-GlcNAcylation.

**Availability and implementation:**

The entire code, data, features used in this study are available in the GitHub repository: https://github.com/pseokyoung/o-glcnac-prediction

## 1 Introduction

O-GlcNAcylation, an essential post-translational modification, involves the attachment of O-linked N-acetylglucosamine (O-GlcNAc) to the serine or threonine residues of proteins. This modification is critical for sensing the nutritional status of cells and extensively regulates cellular functions through interactions with various proteins. Aberrant O-GlcNAcylation is closely associated with various diseases such as cancer, diabetes, and neurodegenerative disorders, positioning O-GlcNAcylation as a strategic target for the development of treatment and prevention strategies (Fardini *et al.* 2013). Accurate prediction of O-GlcNAcylation sites plays a critical role in elucidating the biological pathways implicated in these conditions, thereby enabling researchers to explore disease mechanisms more thoroughly and to innovate effective treatments (Rocamora *et al.* 2023).

Unlike N-glycosylation, which has a defined sequence motif (Asn-X-Ser/Thr, with X being any amino acid except proline), O-GlcNAcylation does not possess a consistent consensus sequence, making prediction more challenging (Gupta and Brunak 2001). While experimental techniques can precisely detect these sites, they are often expensive and time-consuming. Consequently, researchers are actively focusing on computational approaches that leverage amino acid sequences for more efficient predictions. These computational methods offer a faster and more cost-effective alternative to traditional experimental approaches, significantly accelerating O-GlcNAcylation research (Jochmann *et al.* 2014).

Machine learning (ML) models are achieving notable success in computational approaches to predicting glycosylation sites using amino acid sequences. For instance, (Jia *et al.* 2018) introduced O-GlcNAcPRED-II, an ensemble model that utilizes advanced sampling techniques for high-precision glycosylation site prediction. (Mauri *et al.* 2021) suggested enhancing ML models by incorporating the physicochemical properties of protein sequences, using algorithms such as random forests, gradient boosting trees, and support vector machines to improve prediction accuracy. Additionally, (Akmal *et al.* 2021) developed an artificial neural network (ANN)-based predictor, iGlycoS-PseAAC, integrating Pseudo Amino Acid Composition and positional characteristics, achieving high accuracy in glycosylation site prediction.

Previous studies primarily focused on the sequential properties of proteins, with some consideration of structural properties. However, these approaches were insufficient for capturing the complex spatial arrangements and interactions of amino acids (Caragea *et al.* 2007). The secondary and tertiary structures of proteins significantly influence protein interactions, essential for a comprehensive understanding of glycosylation (Li *et al.* 2016). While some models, such as SPRINT-Gly (Taherzadeh *et al.* 2019), have attempted to incorporate both structural and sequence-based features, they still do not fully account for the influence of neighboring amino acids. Moreover, traditional ANN algorithms in these models have limitations in capturing the sequential nature of protein data. In contrast, more advanced technologies such as recurrent neural networks (RNNs) and convolutional neural networks show superior performance in processing sequential information, demonstrating their effectiveness in various fields (Chen *et al.* 2019, Alkuhlani *et al.* 2022, Hu *et al.* 2024, Seber and Braatz 2024).

Despite their enhanced performance in prediction, their “black box” nature complicates the understanding of how specific features contribute to O-GlcNAcylation, presenting obstacles in deciphering the prediction mechanisms (Lamy and Tsopra 2019). Recent advances in ML, particularly by Transformer-based and pretrained protein language models (PLMs), have shown remarkable success in predicting protein functions and PTM sites (Abramson *et al.* 2024). These models encode amino acids in protein sequences as tokens, allowing them to capture complex patterns and learn meaningful representations from extensive protein data (Qiao *et al.* 2022). Embeddings from PLMs not only enhance the accuracy of specific PTM predictions (Pakhrin *et al.* 2024), but also elucidate sequence motifs and help analyze the mutation effects near PTM sites (Shrestha *et al.* 2024), contributing to the development of interpretable ML models. Therefore, research towards enhancing model interpretability is needed for offering directions to deepen our understanding of mechanisms and the structural and functional context of proteins.

This study aims to enhance the prediction of O-GlcNAcylation sites by leveraging the three-dimensional (3D) spatial information of proteins and to identify key contributing factors through the development of an interpretable ML model. The contributions of this approach include:

1. **Sequential Data Utilization:** Employing RNNs, such as long short-term memory (LSTM) networks, to capture effectively the sequential nature of protein amino acid sequences. This approach enables precise modeling of protein sequence information.
2. **Local Environmental Features Integration:** By examining the environmental context, such as solvent accessibility and the physicochemical properties of neighboring amino acids around the target site, the model incorporates novel structural features. This approach significantly enhances prediction accuracy by providing a more comprehensive view of the site’s context.
3. **Model Interpretability Enhancement:** Implementing sparse neural networks using weight regularization techniques such as Lasso ($L_{1}$) or sparse group Lasso (SGL) to improve the model’s interpretability. This method identifies features crucial to O-GlcNAcylation, thereby offering a deeper understanding of its mechanisms.

Through these approaches, the goal is to not only improve the prediction accuracy of O-GlcNAcylation sites but also shed light on the complex mechanisms underlying protein interactions and glycosylation.

## 2 Materials and methods

### 2.1 Data source and preprocessing

For the development of an O-GlcNAcylation site prediction model, we utilized the O-GlcNAcome database (Wulff-Fuentes *et al.* 2021). This database contains detailed information on over 5000 mammalian proteins and more than 7000 verified O-GlcNAcylation sites. From this comprehensive collection, we randomly selected and refined a dataset that includes 104 proteins, encompassing 428 positive samples and 10 932 negative samples, which is sufficient for our research purposes. This dataset shows a notable imbalance between the two classes, positive or negative for O-GlcNAcylation, with a ratio of ∼1:26. To facilitate effective training of the ML models, we implemented preprocessing techniques for both categorical and continuous data types.

One-hot encoding is a method that converts categorical data into a binary vector format. The method creates new columns for each category, with binary values indicating the presence or absence of category attributes, making it easier for ML models to process the data. Additionally, we normalized continuous data to ensure all values fall within a uniform scale from 0 to 1, a process known as data scaling. This process is crucial for minimizing disparities among features, thus enhancing the efficiency and effectiveness of model training.

These preprocessing steps ensure the data are in the best possible format, significantly improving the quality of the dataset for model training.

### 2.2 Local environmental features

**Figure 1.**
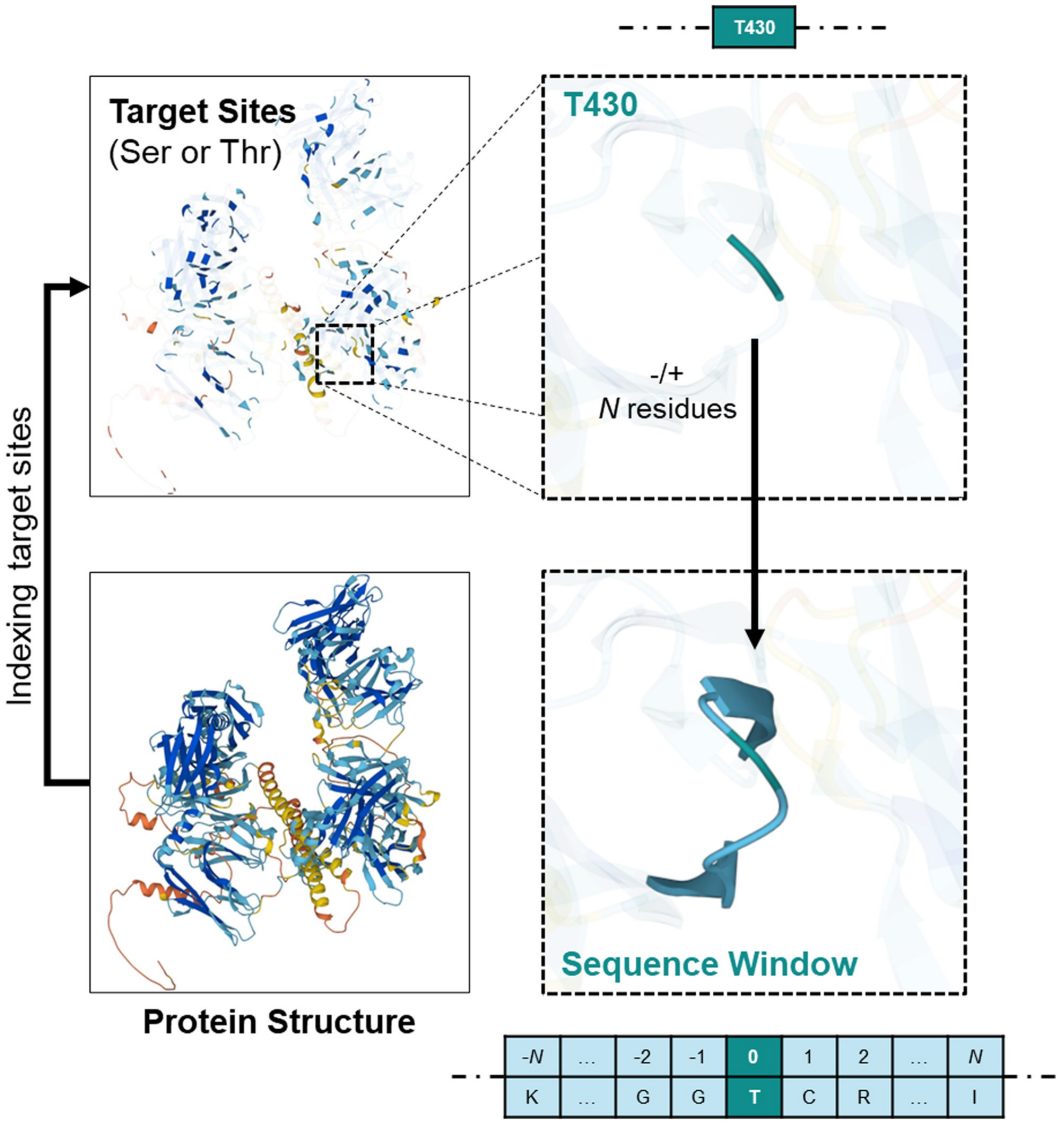
Sequence window from the target site in proteins. For every target site, ± N amino acids from the site are included to construct the sequence window.

To identify glycosylation sites, as shown in Fig. 1, the target site is defined as the location of interest with serine or threonine residues. Surrounding these sites, sequence windows are established to incorporate *N* amino acids both before and after the target site. *N* value is set to 10 to analyze the sequential context of adjacent amino acids.

The features at every position of sequence window influences model performance. Thus, extracting meaningful attributes from amino acid sequences to construct informative sequence windows is crucial for building accurate and reliable ML models. In addition to these sequence windows, we have found that structural features are essential for predictive accuracy.

**Figure 2.**
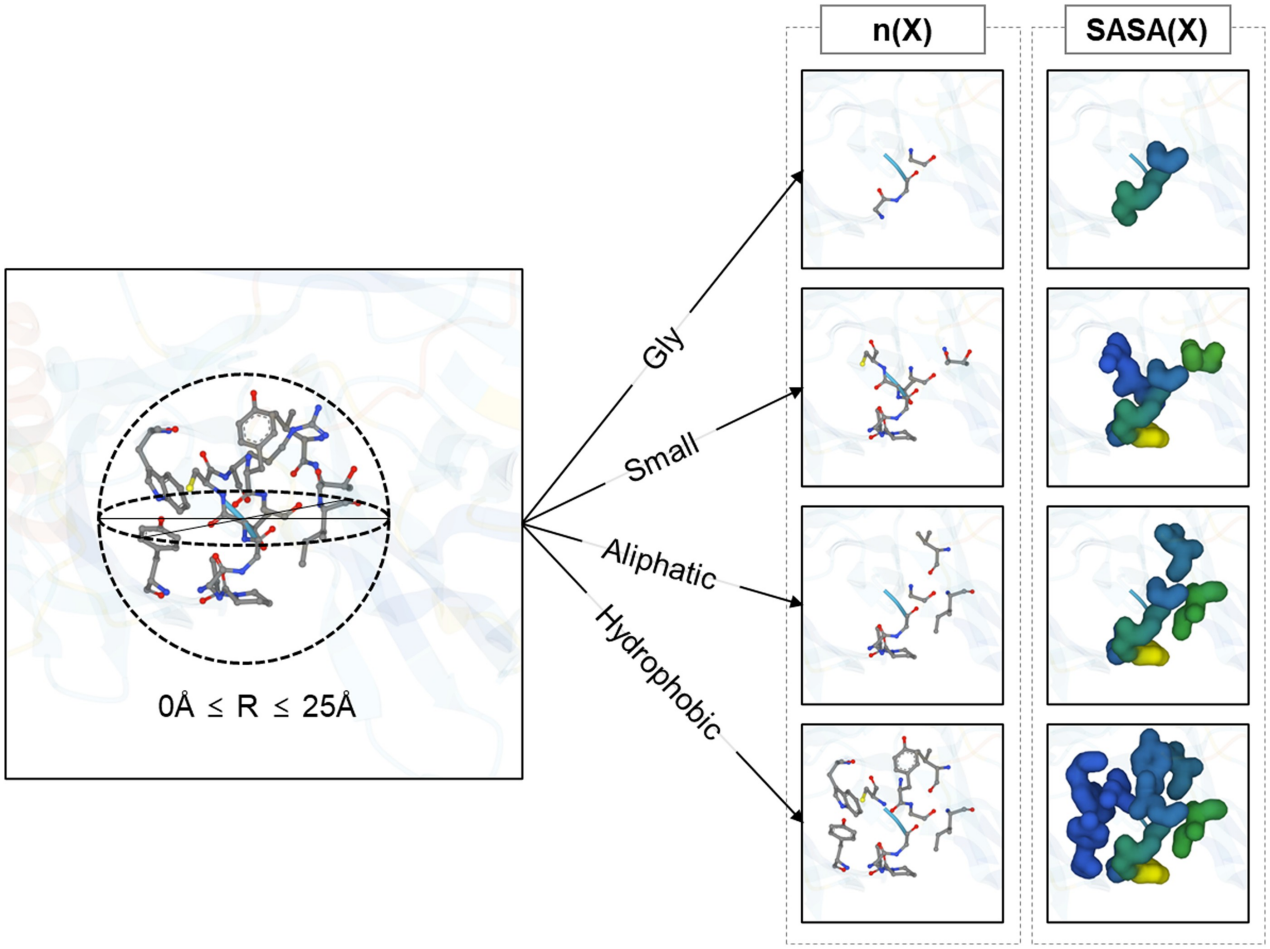
A graphical representation of an amino acid residue and its immediate local surroundings, defined by various cutoff distances. Features are computed for the residue as a standalone entity (at a 0 Å radius) and in relation to its neighboring residues situated within radial cutoffs of 5, 10, 15, 20, and 25 Å.

In Fig. 2, we introduce “local environmental features,” novel attributes aimed at a detailed analysis of the surrounding environment. The local environment is depicted as a 3D space, with a radius ranging from 0 to 25 angstroms (Å). At a minimum radius of 0 Å, the focus is limited to the centered position itself. As the radius expands, it includes more neighboring amino acids and their characteristics. This approach allows for calculating the distribution and total solvent-accessible surface area (SASA) of neighboring amino acids based on their physicochemical properties, such as hydrophobicity, size, polarity, and charge. The total SASA of aliphatic amino acids or the number of nonpolar amino acids are examples of such features (see more details in Table S1, available as supplementary data at *Bioinformatics* online). To perform these calculations, we utilized Computed Structure Models from the RCSB Protein Data Bank (Berman *et al.* 2000), assessing structural features. Following the modeling phase, we proceeded to generate the coordinates, incorporating hydrogen atoms into each structure. The PSFGEN plugin facilitated this process within the Visual Molecular Dynamics software (Humphrey *et al.* 1996). Additionally, we determined the partial charges assigned to each atom by utilizing the CHARMM36m force field (Klauda *et al.* 2010, Huang *et al.* 2017).

### 2.3 Sparse recurrent neural networks

**Figure 3.**
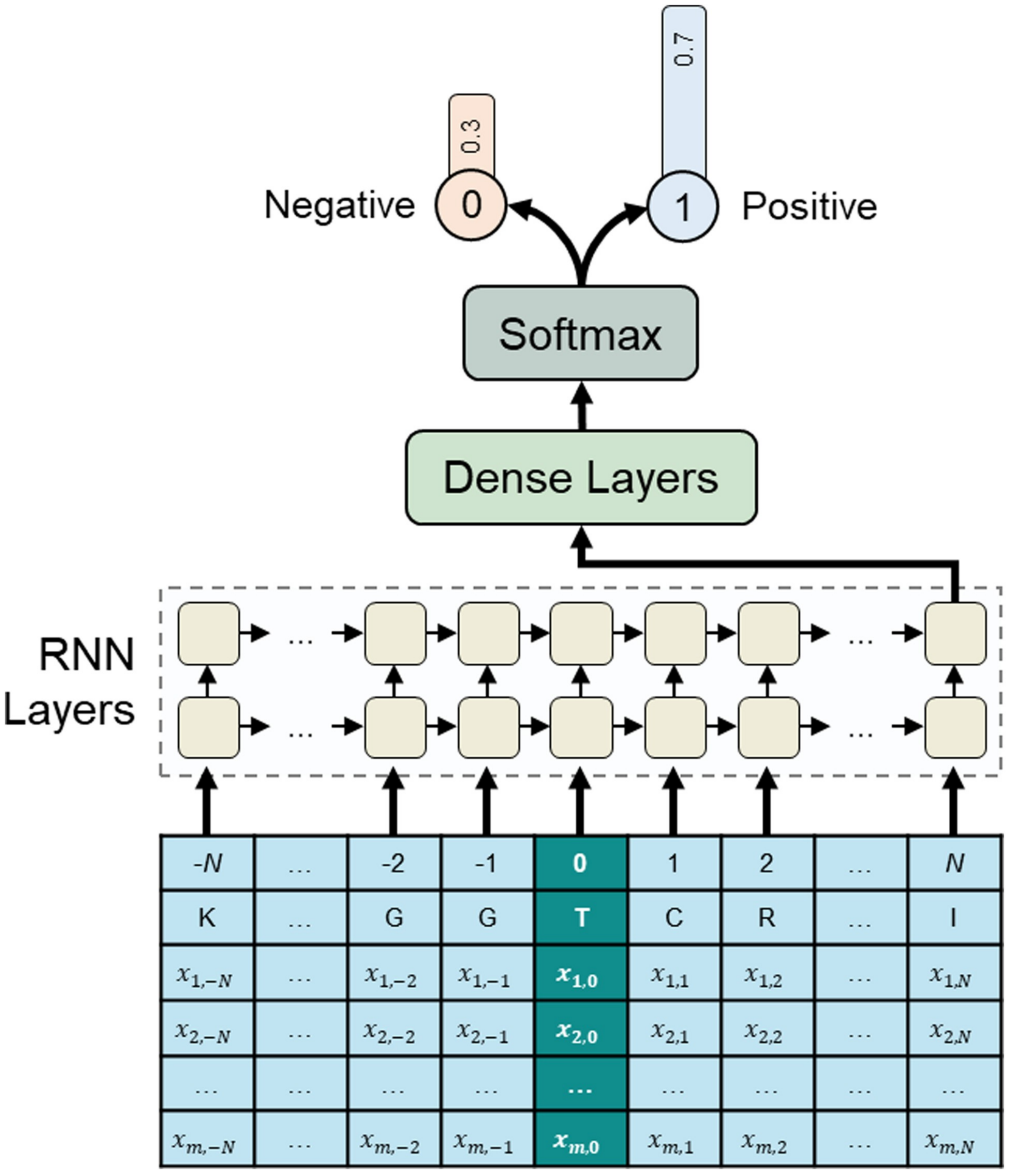
An architecture of the RNN leveraging a sequence window to predict O-GlcNAcylation likelihood. The target site is located at the center of an input sequence with neighboring amino acids. Input data has dimensions $\left(m,N\right)$, where *m* is the number of input features and N is the number of adjacent residues on either side of the target site. The output is the probability of the target site being O-GlcNAcylated.

ANN models excel modeling the complex and nonlinear relationship between input data and output labels. Among these, the Multilayer perceptron (MLP) represents a fundamental form, where input data passes through multiple fully connected layers to produce output labels. However, MLPs have limitations in handling sequential information, such as time series data or protein sequences, which contain continuous information. To overcome these limitations, RNNs are employed as shown in Fig. 3. RNNs efficiently process time series data, managing continuous information adequately by sequentially processing feature vectors at each time step. The recurrent unit of an RNN takes the hidden state from the previous step and the current step’s feature vector as inputs to compute the current hidden state. Through this process, sequential information continuously passes through the recurrent unit, and the hidden state output at the last step of the RNN layer contains the sequence’s core information, referred to as the context vector. This context vector is then transformed into the probability of glycosylation occurrence at the target site, passing through subsequent dense layers and a softmax function.

To enhance model efficiency, weight regularization is applied to implement sparse networks. Sparsity strengthens by reducing connections between nodes within the network or diminishing the size of nodes. This process assists the model in filtering out unnecessary information, focusing on more critical features. It yields the effect of feature selection in input groups and pruning in hidden groups. Such an approach contributes to simultaneously improving the network’s efficiency and performance. To ensure the model’s sparsity, a regularization term is added to the loss function, defining the optimal parameters by


(1) $$W^{*}=\arg\underset{W}{\min}\left\{L\left(y,\;\hat{y}\right)+\lambda R\left(W\right)\right\}$$

In the process of defining the optimal parameters with weight regularization, all trainable parameters within the network are concatenated into a column vector denoted by $W\in R^{Q}$, where $W^{*}$ represents the parameters optimized through weight regularization. The loss function L uses the cross-entropy function for binary labels (Ho and Wookey 2020), with $y_{i}^{\left(j\right)}$ and ${\hat{y}}_{i}^{\left(j\right)}$ representing the actual label value for the input vector x and the predicted value calculated through the neural network, respectively. The regularization term R adjusts the balance between the two terms through the scalar coefficient $\lambda \in R^{+}$.

Weight regularization can involve Lasso ($L_{1}$) or SGL regularization. These regularization methods are equivalent in computational complexity, represented as $O\left(Q\right)$, where Q denotes the number of parameters. $L_{1}$ regularization operates by imposing a penalty based on the absolute values of parameters in the loss function,


(2) $$R_{l1}\left(W\right)={\left\|W\right\|}_{1}$$

SGL regularization ensures sparsity among the remaining connections after group-level node removal, implementing group sparsity regularization as (Scardapane *et al.* 2017):


(3) $$R_{\mathrm{SGL}}\left(W\right)=\alpha \sum_{l\in S}\sum_{k=1}^{N_{l}}\sqrt{w_{k}^{\left(l\right)}}{\left\|w_{k}^{\left(l\right)}\right\|}_{2}+\left(1-\alpha \right){\left\|W\right\|}_{1}$$

where the weight vector $w_{k}^{\left(l\right)}$ is the kth column vector in the lth layer of the network, which contains $N_{l}$ neurons; S is a subset of all layer groups $G=\left\{1,2,\ldots ,H+1\right\}$, including the input layer (l=1) and H hidden layers (2≤H≤H+1); and α adjusts the balance between group Lasso and Lasso regularization.

This process optimizes network performance while managing model complexity and preventing overfitting. $L_{1}$ regularization reduces certain parameter weights to 0, serving as feature selection, whereas SGL regularization promotes group-level sparsity, enabling feature selection on a broader scale. These regularization methods encourage the network to reduce unnecessary connections and focus on more critical connections.

### 2.4 Model evaluation metrics

In classification problems, the accuracy metric is often used to evaluate model performance. Accuracy is determined by four elements: True Positives (TP), False Positives (FP), True Negatives (TN), and False Negatives (FN). These elements calculate the ratio of correctly classified samples among all samples to represent model performance. The formula for accuracy is:


(4) $$\mathrm{accuracy}=\frac{\mathrm{TP}+\mathrm{TN}}{\mathrm{TP}+\mathrm{TN}+\mathrm{FP}+\mathrm{FN}}$$

When data are imbalanced, accuracy can provide an overly optimistic evaluation for the majority class, becoming an unreliable indicator. In such cases, the $F_{1}$ score is used as a primary performance metric. The $F_{1}$ score, independent of the number of samples correctly classified as negative, is calculated using the harmonic mean of precision and recall. The formulas for these metrics are (Chicco and Jurman 2020):


(5) $$\mathrm{precision}=\frac{\mathrm{TP}}{\mathrm{TP}+\mathrm{FP}}$$

(6) $$\mathrm{recall}=\frac{\mathrm{TP}}{\mathrm{TP}+\mathrm{FN}}$$

(7) $$F_{1}=2\frac{\mathrm{precision}\times \mathrm{recall}}{\mathrm{precision}+\mathrm{recall}}$$

These metrics ensure the model does not overlook the importance of the minority class, especially in imbalanced data, providing a more accurate evaluation of model performance. The $F_{1}$ score is useful for assessing how well a model identifies positive samples and minimizes misclassification of negative samples, indicating the model’s overall balanced performance. The $F_{1}$ score ranges from 0% to 100%, where a higher score indicates better performance. A high $F_{1}$ score close to 100% suggests the model has low rates of FPs and FNs. In our current study, we have significantly improved the $F_{1}$ score, achieving a multiple-fold increase compared to previous studies.

### 2.5 Statistical analysis: ratio of mean feature values

In addition to deriving important features from the sparse recurrent neural networks (SRNN) model, a statistical analysis is also performed on the identified key features. By comparing the mean feature values between positive and negative samples, we aimed to find statistically significant differences between the two groups. The ratio for each feature between the two groups is calculated from:


(8) Ratiox=x¯posx¯neg, for x¯pos≥x¯neg-x¯negx¯pos, for x¯neg≥x¯pos

where ${\bar{x}}_{\mathrm{pos}}$ represents the mean feature value of positive samples, and ${\bar{x}}_{\mathrm{neg}}$ is that of negative samples. This ratio provides a straightforward interpretation: a higher positive ratio indicates that the feature is favorable for the target site to be O-GlcNAcylated, while a higher negative ratio suggests the opposite. This understanding helps us better comprehend the features that promote or inhibit the modification.

## 3 Results

### 3.1 Comparative analysis of predictive performance

To evaluate the effectiveness of our proposed features and the use of RNN models in predicting O-GlcNAc glycosylation sites, we compared our models against existing glycosylation site prediction tools. The benchmark models selected for comparison are DictyOGlyc 1.1 (Gupta *et al.* 1999), YinOYang 1.2 (Gupta and Brunak 2001), O-GlcNAcPRED-DL (Hu *et al.* 2024), and the MLP model proposed by (Mauri *et al.* 2021).

Our RNN models employ LSTM algorithms with three different feature sets: primary amino acid sequences (Primary-LSTM), structural dynamic features (Secondary-LSTM), and local environmental features (Local-LSTM). For model training, the data were split into 80% training data and 20% test data, and the optimal hyperparameters for each ML model were determined through five-fold cross-validation. Details on the models’ specific structures and designs can be found in Table S2, available as supplementary data at *Bioinformatics* online. To prevent overfitting, the early stopping technique was used (Ying 2019) and, for parameter updates, the Adam optimization algorithm with a learning rate of 0.001 was used. Finally, the generalized prediction performance of the structurally optimized models was evaluated and compared using the Monte-Carlo cross-validation method, repeating the process five times (Xu *et al.* 2004). To address the issue of data imbalance in each training dataset, we replicate positive samples to match the quantity of negative samples, thereby effectively minimizing training bias.

**Figure 4.**
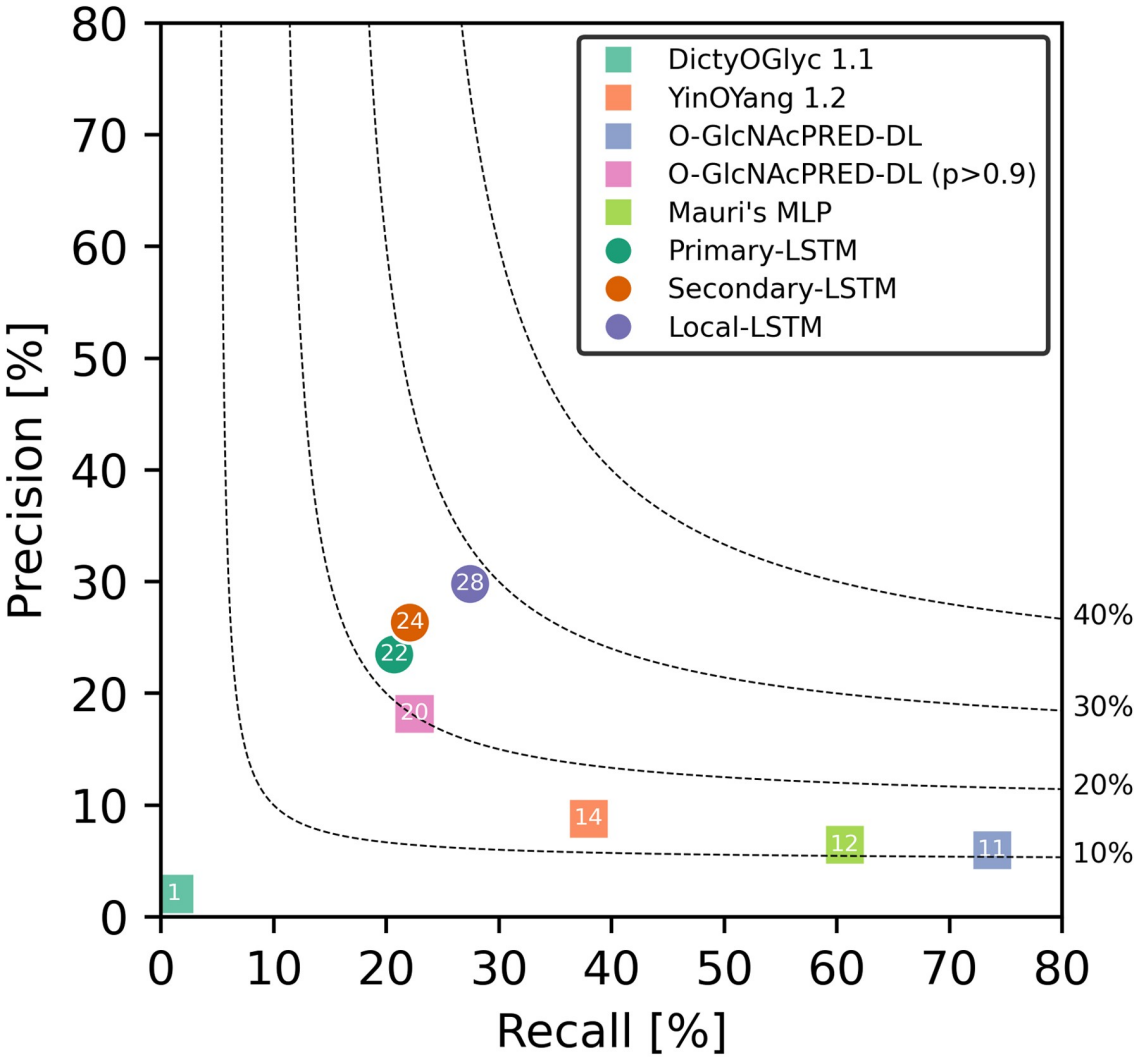
Performance of various ML models for O-GlcNAcylation site prediction in terms of precision and recall. The benchmark models from the literature are represented by squares, while our LSTM models are depicted by circles. Dotted lines indicate different $F_{1}$ scores: 10%, 20%, 30%, and 40%. Each shape is annotated with its corresponding $F_{1}$ score.

Figure 4 compares the glycosylation site prediction performance of the benchmark models with our LSTM models. The four benchmark models demonstrated $F_{1}$ scores below 15%, except for the O-GlcNAcPRED-DL model, which achieved an $F_{1}$ score of 20.1% when the threshold was set to 0.9. These models exhibited relatively higher recall scores compared to precision scores, indicating a tendency to over-classify sites as glycosylated. This bias results in a higher number of false positives, meaning more non-glycosylated sites are incorrectly labeled as glycosylated, which can be time-consuming to investigate experimentally. Adjusting the threshold in the O-GlcNAcPRED-DL model to 0.9 reduced the number of predicted positive sites to include only those with high probabilities, thereby balancing precision and recall and improving the $F_{1}$ score.

In contrast, our LSTM models with different feature sets all achieved $F_{1}$ scores above 20%. The Primary-LSTM model showed an $F_{1}$ score of 21.9%, the Secondary-LSTM model achieved 24.0%, and the Local-LSTM model recorded the highest $F_{1}$ score of 28.3%, ∼1.4 times higher than that of the best benchmark model. The superior performance of the Local-LSTM model can be attributed to its ability to consider the spatial context of interactions between residues around the target site and solvents. By capturing important biochemical factors such as steric hindrance and electrostatic interactions, the model more accurately predicts glycosylation sites.

Despite this improvement, there is still considerable room for further enhancement. Addressing current limitations and exploring alternative approaches can improve predictive accuracy. First, expanding the dataset in both size and diversity would provide more examples, enabling models to better learn the patterns of glycosylation sites. Second, while RNNs are effective for capturing protein sequence information, they may not be the optimal architecture for modeling spatial relationships between neighboring amino acids. Exploring alternative ML methods, such as graph neural networks, could help directly capture this spatial information. Finally, applying pretrained PLMs, which are state-of-the-art technology, could also improve accuracy. These suggestions for future research could advance the field of protein PTM site prediction.

### 3.2 Feature selection for improving model efficiency and transparency

In the previous section, we demonstrated that incorporating local environmental features enhances the accuracy of glycosylation site prediction. However, due to the “black box” nature of ANNs, it remains challenging to precisely identify which specific features contribute most to the improved predictive performance. Therefore, from this section, we aim to identify the most influential features among 498 local spatial features by employing a feature selection approach based on SRNN models described in Section 2.3.

In this framework, SRNNs with regularization techniques, such as $L_{1}$ and SGL, serve as effective tools for feature selection. As a result, features associated with higher weight values are considered more important, as they have greater influence on predictions. Selecting features through regularization not only simplifies the feature set but can also potentially enhance both the interpretability and generalizability of the model.

To evaluate the effectiveness of this feature selection method, we ranked the features based on their weight values derived from the SRNN models. Starting from the highest-weighted features, we incrementally added features in order of importance and measured the model’s predictive performance, specifically the $F_{1}$ score, as a function of the fraction of top-ranked features selected. Effective feature selection should enable the model to maintain or even improve predictive performance using only a reduced subset of features, making the model more efficient and easier to interpret.

**Figure 5.**
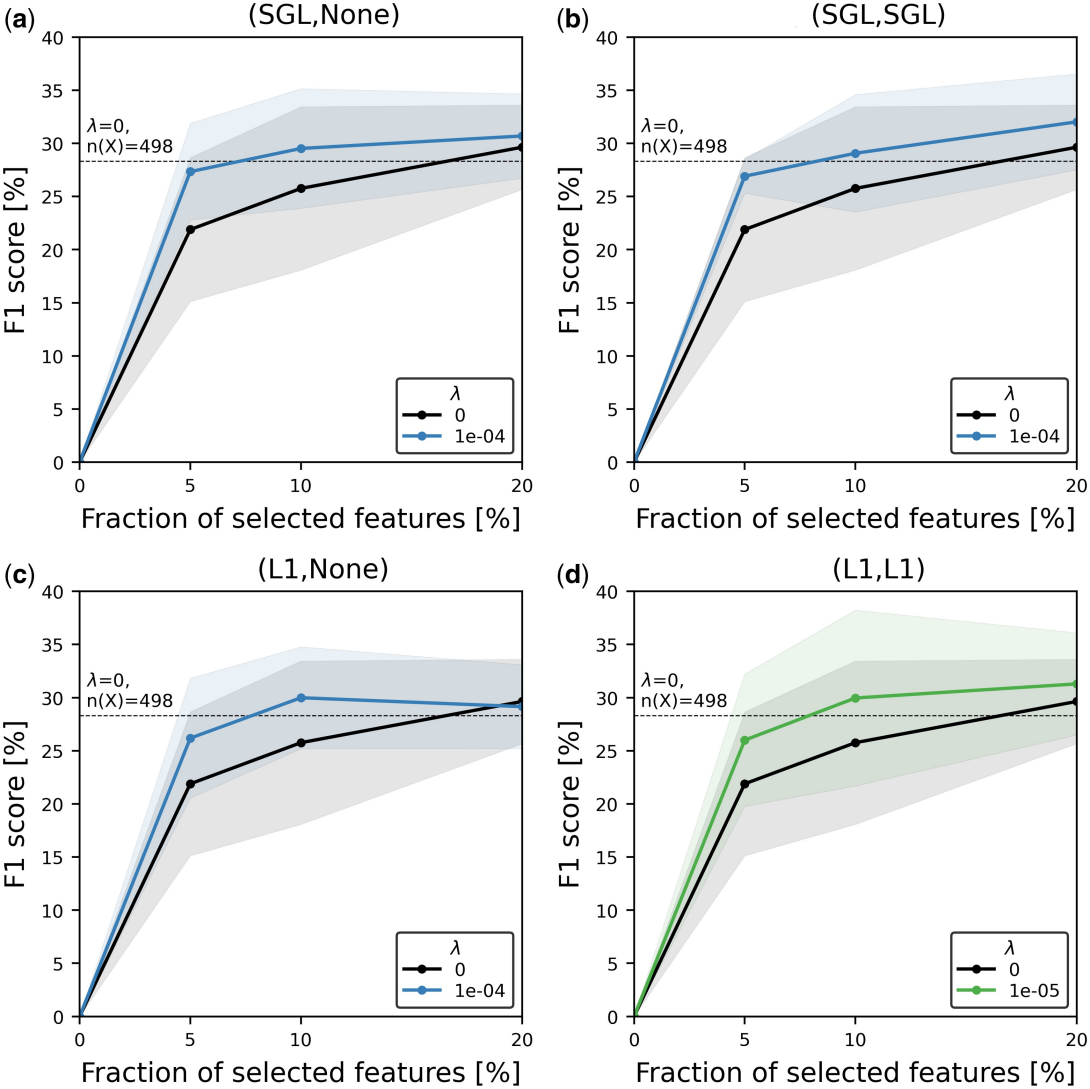
$F_{1}$ scores across the fraction of selected top-ranked features, chosen based on the optimal lambda value. In the subtitles, (Input, Hidden) indicates whether regularization is applied to the input and hidden groups, respectively, with either the $L_{1}$ or SGL regularization method. The black solid line represents the model without regularization, and the dotted line indicates the $L_{1}$ score of the model using all features without any regularization.

As shown in Fig. 5, our results indicate that models with effective feature selection achieved high $F_{1}$ scores using only a small fraction of the top-ranked features. For example, when using just the top 10% of features, all models with regularization outperformed both the non-regularized model (solid black line) and the baseline model that used all 498 features (dotted line).

In the subtitles in Fig. 5, different regularization configurations are indicated; e.g. when only SGL regularization is applied to the input group, it is labeled as (SGL, None). Among the four configurations tested, the (SGL, SGL) model achieved the highest $F_{1}$ score of 32.02% using the top 20% of features. This significantly reduces the number of features from 498 to just 100, which not only simplifies the model but also improves its performance. These results underscore the SRNN model’s capability to effectively prioritize informative features, leading to better performance with fewer inputs. By identifying and using the most informative features, we can build models that are both efficient and transparent, facilitating better understanding and potential application in related fields.

### 3.3 Statistical analysis of selected features

**Figure 6.**
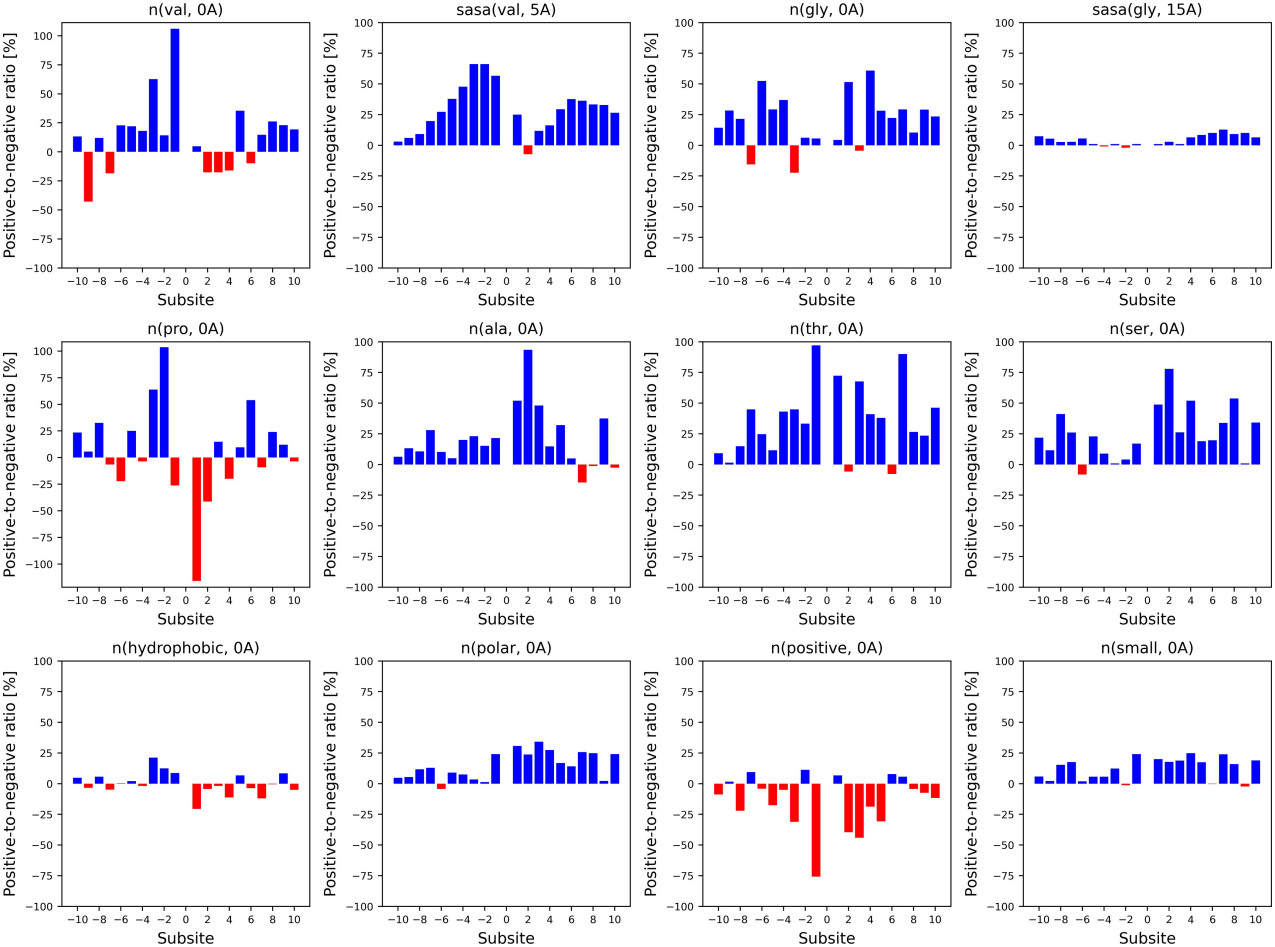
The ratio of the mean feature values between positive and negative sample across subsite locations from −10 to +10 for key features. Positive ratios (upwards) indicate that the feature value is higher in positive samples than in negative samples, while negative ratios (downwards) show the opposite situation.

This section investigates the validation of the top 12 features selected from a total of 498 local environmental features and their biological significance in predicting O-GlcNAcylation sites. These features were consistently selected across all model configurations tested in Section 3.2. The enzyme O-GlcNAc transferase (OGT) catalyzes the modification, and understanding the amino acid context surrounding O-GlcNAcylation sites is crucial for elucidating the mechanisms governing OGT’s substrate specificity. Figure 6 illustrates their statistical analysis based on the dataset described in Section 2.5.

The presence of valine (Val), glycine (Gly), and alanine (Ala) near O-GlcNAcylation sites significantly enhances the probability of this PTM occurring (Wu *et al.* 2014, Li *et al.* 2016, Chong *et al.* 2023). These amino acids have small, non-polar side chains, which are believed to reduce steric hindrance and increase the SASA of the target residues. This makes it easier for OGT to bind to its substrate. Proline (Pro), particularly when located at the −2 and −3 positions relative to the modification site, plays a unique role by disrupting local secondary structures such as alpha-helices and beta-sheets (Jochmann *et al.* 2014). This disruption leads to increased conformational flexibility around the modification site, making it easier for OGT to access and modify the target residues.

In alignment with previous studies (Jochmann *et al.* 2014, Wu *et al.* 2014, Maynard and Chalkley 2021), an abundance of serine (Ser) and threonine (Thr) residues near the target site also increases the likelihood of O-GlcNAcylation. When multiple Ser or Thr residues are clustered together, the probability that one or more will be accessible for modification by OGT increases. Conversely, the presence of positively charged amino acids near target sites is generally unfavorable for O-GlcNAcylation. Their positive charges can cause electrostatic repulsion or steric hindrance, making it more difficult for OGT to access the target residues. However, when these positively charged residues are specifically located at the −2 and +1 positions, they might interact beneficially with OGT or stabilize the enzyme-substrate binding, thereby facilitating the modification (Hansen *et al.* 1998, Wu *et al.* 2014).

Hydrophobic residues located before the target site (subsites −1, −2, and −3) may stabilize the local protein structure, helping to position the Ser or Thr residues in a conformation favorable for OGT recognition and binding. In contrast, when hydrophobic residues are found after the target site (subsites +1 to +4), they might cause steric hindrance or form a compact, hydrophobic pocket that blocks OGT’s access to the target residues. Polar amino acids, due to their side chains interacting with water, can increase the solvent exposure of target residues. They may also form hydrogen bonds or electrostatic interactions with OGT, stabilizing the enzyme’s binding to the protein and promoting efficient modification.

These findings highlight the complex interactions between specific amino acids and their spatial arrangement in modulating O-GlcNAcylation, a critical PTM. Understanding these patterns not only reinforces the consistency and validity of existing findings within the broader scientific context but also helps in predicting potential O-GlcNAcylation sites and deciphering OGT’s substrate specificity.

## 4 Conclusion

This study proposes an approach to enhance O-GlcNAcylation site prediction by introducing local environmental information and utilizing the capabilities of SRNN. The approach establishes a sequence window around target sites, which includes neighboring amino acids in sequence, further enriched with the spatial context from the 3D structure of the protein. We demonstrate that the SRNN is able to effectively capture the sequential context of proteins, while its architecture enables identifying key factors that improve model performance. A model utilizing only the top 20% of features outperforms a full-feature model by 13%, achieving a minimum 1.4-fold increase compared to existing PTM models. These findings highlight the effectiveness of our method and its ability to incorporate spatial information and to selectively identify significant features for O-GlcNAcylation prediction.

## Supplementary Material

btaf034_Supplementary_Data

## Data Availability

The entire code, data, features used in this study are available in the GitHub repository: https://github.com/pseokyoung/o-glcnac-prediction
