# Correction to: Enhanced O-glycosylation site prediction using explainable machine learning technique with spatial local environment

**DOI:** 10.1093/bioinformatics/btaf281

**Published:** 2025-05-10

This is a correction to: Seokyoung Hong, Krishna Gopal Chattaraj, Jing Guo, Bernhardt L Trout, Richard D Braatz, Enhanced O-glycosylation site prediction using explainable machine learning technique with spatial local environment, *Bioinformatics*, Volume 41, Issue 2, February 2025, https://doi.org/10.1093/bioinformatics/btaf034

The following changes have been made to the originally published manuscript. The **Funding** section has been emended to read additionally: “Seokyoung Hong was supported by Korea Institute for Advancement of Technology (KIAT) grant (P0017304, Human Resource Development Program for Industrial Innovation) funded by the Ministry of Trade, Industry and Energy (MOTIE), Republic of Korea.”

