# Supplementary material for: Enhanced O-glycosylation site prediction using explainable machine learning technique with spatial local environment
Source: Bioinformatics. 2025 Jan 29;41(2):btaf034. doi: 10.1093/bioinformatics/btaf034 (PMC11814488; doi:10.1093/bioinformatics/btaf034)
Supplement: btaf034_Supplementary_Data [file btaf034_supplementary_data.zip › d3337_Supplementary Information.docx]

**Supplementary Information**

**Table S1.** In this study, augmented structural features were calculated not only for individual residues (0 Å) but also in conjunction with their neighboring residues, employing cutoff distances ranging from 5 to 25 Å. For the first 21 features, the focus was on quantifying the number of specific amino acids and their solvent-accessible surface area (SASA), providing a detailed analysis of both the residue composition and their spatial accessibility within the specified range. The study presented a comprehensive set of 498 features to encompass a broad spectrum of structural characteristics. This extensive feature set was meticulously selected to more accurately capture the physicochemical phenomena relevant to the current investigation, providing a robust foundation for analyzing and understanding the intricate molecular interactions at play.

| # | Feature name | Note | # | Feature name | Note |
| --- | --- | --- | --- | --- | --- |
| 1 | Hydrophobic | from VMD | 15 | Negatively charged polar | From Mauri et al. |
| 2 | Hydrophilic | SER, THR, ASN, GLN, TYR, LYS, ARG, HIS, ASP, GLU | 16 | Non-polar sulfur containing |  |
| 3 | Polar | SER, THR, ASN, GLN, TYR | 17 | Non-polar aromatic |  |
| 4 | Aromatic | from VMD | 18 | All individual amino acids | 20 amino acids |
| 5 | Aliphatic | from VMD | 19 | SASA of target residues |  |
| 6 | Charged | HIS, LYS, ARG, GLU, ASP | 20 | SASA of backbone of residues |  |
| 7 | Positive | ARG, HIS, LYS | 21 | SASA of sidechain of residues |  |
| 8 | Negative | ASP, GLU | 22 | Net charge of residues |  |
| 9 | Very small | From Mauri et al. | 23 | Side-chain charge |  |
| 10 | Small |  | 24 | Backbone charge |  |
| 11 | Normal |  | 25 | Net charge of exposed residues |  |
| 12 | Long |  | 26 | Net charge of exposed backbone |  |
| 13 | Polar uncharged with hydroxyl group |  | 27 | Net charge of exposed sidechain |  |
| 14 | Polar uncharged with amide |  |  |  |  |

**Table S2.** Hyperparameter setting of different ML models.

| Model | Input shape | Output shape | Recurrent unit | Dense unit |
| --- | --- | --- | --- | --- |
| Mauri’s MLP | (73) | (2) | - | 5 layers, 64 nodes each |
| Primary-LSTM | (21, 20) | (2) | 3 layers, 64 nodes each | 5 layers, 64 nodes each |
| Secondary-LSTM | (21, 53) | (2) | 4 layers, 64 nodes each | 4 layers, 64 nodes each |
| Local-LSTM | (21, 498) | (2) | 5 layers, 64 nodes each | 3 layers, 64 nodes each |
